# The Challenge of Diagnosing Scirrhous Gastric Cancer by Endoscopic Biopsy: A Case Report

**DOI:** 10.7759/cureus.87334

**Published:** 2025-07-05

**Authors:** Yuka Ikeda, Masaya Iwamuro, Tadashi Yoshino, Takehiro Tanaka, Nobumasa Ikeda, Motoyuki Otsuka

**Affiliations:** 1 Department of Internal Medicine, Clinic Ikeda, Kan'onji, JPN; 2 Department of Gastroenterology and Hepatology, Okayama University Graduate School of Medicine, Dentistry, and Pharmaceutical Sciences, Okayama, JPN; 3 Department of Pathology, Okayama University, Okayama, JPN; 4 Department of Pathology, Okayama University Hospital, Okayama, JPN

**Keywords:** endoscopic biopsy, esophagogastroduodenoscopy, immunohistochemistry, linitis plastica, scirrhous gastric cancer

## Abstract

Scirrhous gastric cancer, also known as linitis plastica, is a rare and aggressive subtype of gastric carcinoma that poses significant diagnostic challenges due to its submucosal infiltration and often normal-appearing mucosa. We report a case involving a 30-year-old Japanese woman who presented with a six-month history of epigastric pain and postprandial vomiting. Initial endoscopic examination revealed erythema and mucosal swelling, with limited antral distensibility and resistance during duodenal intubation. Despite 12 mucosal biopsies, histopathological examination revealed no evidence of malignancy. Given the strong clinical and endoscopic suspicion of scirrhous gastric cancer, additional deep sections and immunohistochemical staining were performed. These revealed scattered signet-ring cell carcinoma and poorly differentiated adenocarcinoma, with positive immunostaining for p53 and Ki67. The patient underwent total gastrectomy, and the diagnosis of scirrhous gastric cancer was confirmed on the resected specimen. This case highlights the importance of a high index of clinical suspicion, close collaboration between endoscopists and pathologists, and the utility of ancillary diagnostic tools, such as immunohistochemistry, in identifying subepithelial gastric malignancies that may be missed on conventional biopsy.

## Introduction

Scirrhous gastric cancer, also referred to as linitis plastica, is a relatively infrequent but highly aggressive subtype of gastric carcinoma that presents considerable diagnostic challenges [1,2]. Epidemiologically, it accounts for approximately 10-20% of all gastric cancer cases worldwide. Scirrhous gastric cancer is characterized by rapid progression, frequent lymphovascular invasion, and peritoneal dissemination, resulting in poorer survival outcomes compared to other types of gastric cancer. The five-year survival rate for patients with scirrhous gastric cancer is significantly lower than that for those with localized or intestinal-type gastric cancers. Therefore, early diagnosis is particularly critical when this subtype is suspected.

Histopathologically, this subtype is characterized by a diffuse infiltration of poorly differentiated adenocarcinoma or signet ring cell carcinoma, which primarily invades the submucosal and muscular layers of the gastric wall [1,2]. Unlike more localized, polypoid, or ulcerative forms of gastric cancer, scirrhous gastric cancer rarely forms discrete masses or well-demarcated ulcerative lesions. Instead, it induces diffuse thickening and stiffening of the gastric wall, often with minimal or no visible changes to the mucosal surface. Due to this infiltrative growth pattern, the mucosa may appear deceptively normal or show only subtle erythema or flattening on endoscopic examination. Consequently, conventional endoscopic biopsy, which samples only the superficial mucosal layers, often fails to capture malignant cells, resulting in a high rate of false-negative histological diagnoses [3,4]. This discrepancy between the depth of tumor infiltration and the superficial sampling achieved by standard biopsy techniques significantly contributes to diagnostic delays and underrecognition of this disease in its early stages.

In clinical practice, a high index of suspicion is required to detect scirrhous gastric cancer, particularly in cases where endoscopic findings do not fully explain the patient’s symptoms or when imaging studies suggest abnormalities not confirmed by routine biopsy. In such cases, additional diagnostic strategies, including targeted deep biopsies, histological reassessment, and immunohistochemical staining, may be essential for accurate detection and diagnosis.

Here, we report a case of scirrhous gastric cancer in a young female patient who initially presented with non-specific gastrointestinal symptoms. Despite multiple mucosal biopsies showing no evidence of malignancy, the diagnosis was ultimately confirmed through immunohistochemical staining and deeper tissue sectioning. This case highlights the unique diagnostic challenges posed by scirrhous gastric cancer and underscores the importance of comprehensive pathological evaluation when clinical suspicion remains high.

## Case presentation

A 30-year-old Japanese woman with no significant past medical history and no regular medications presented with a six-month history of epigastric pain and occasional postprandial vomiting. At the age of 23, she had undergone a urea breath test for screening purposes, which was negative for *Helicobacter pylori* infection. An esophagogastroduodenoscopy performed at that time had revealed no abnormalities. She had no notable family history of gastric or other malignancies. Her epigastric pain and occasional postprandial vomiting resolved spontaneously, and she remained asymptomatic thereafter without undergoing any screening esophagogastroduodenoscopy.

**Figure 1:**
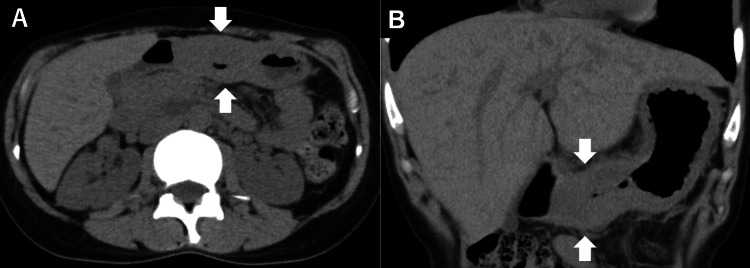
Computed tomography (CT) finding. CT showing marked thickening of the gastric wall, predominantly in the antrum (arrows), suggestive of infiltrative gastric malignancy. Axial (A) and coronal (B) views of the CT scan.

**Figure 2:**
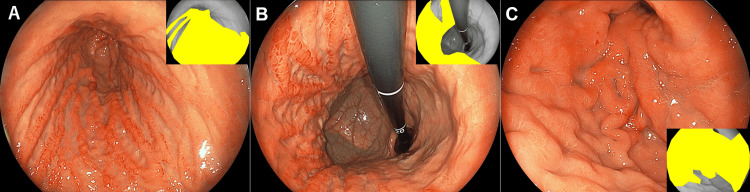
Endoscopic findings of the stomach. Diffuse erythema and edematous mucosal swelling are observed, particularly along the greater curvature (A,B). Poor distensibility of the antrum despite maximal insufflation (C). These findings raised suspicion for scirrhous gastric cancer. The yellow-highlighted areas in the inset images indicate the gastric cancer lesions.

On physical examination, no pallor was observed in the palpebral conjunctivae. Her abdomen was flat and soft, with no palpable masses. No lymphadenopathy was noted. Laboratory tests, including tumor markers such as carcinoembryonic antigen and carbohydrate antigen, showed no abnormalities in hematological or biochemical parameters (Table 1). A contrast-enhanced computed tomography (CT) scan revealed marked thickening of the gastric wall, predominantly involving the antrum (Figure 1, arrows). A subsequent esophagogastroduodenoscopy (EG-760Z; Fujifilm Holdings Corporation, Tokyo, Japan) demonstrated no signs of mucosal atrophy. However, in contrast to the intact mucosa noted seven years earlier, the current examination revealed erythema and swelling of the mucosal folds, particularly along the greater curvature of the stomach (Figure 2A, 2B). The antrum remained minimally distensible despite maximal insufflation (Figure 2C), and passage into the duodenum was achieved with some resistance. Given the prominent submucosal rigidity and mucosal changes, scirrhous gastric carcinoma was strongly suspected. Twelve biopsy specimens were obtained endoscopically from the greater curvature of the mid and lower gastric body.

**Table 1:** Laboratory results on presentation

Blood test parameter	Patient value	Reference range
White blood cells (/μL)	5,100	3,300–8,600
Neutrophil (%)	61.1	40–70
Hemoglobin (g/dL)	11.9	11.6–14.8
Platelets (/μL)	22.7×10^4^	15.8×10^4^–34.8×10^4^
Total protein (g/dL)	7.1	6.6–8.1
Albumin (g/dL)	4.3	4.1–5.1
Creatinine (mg/dL)	0.61	0.46–0.79
Lactate dehydrogenase (U/L)	124	124–222
Sodium (mmol/L)	138	138–145
Potassium (mmol/L)	3.8	3.6–4.8
Chloride (mmol/L)	105	98–107
Aspartate aminotransferase (U/L)	16	13–30
Alanine aminotransferase (U/L)	8	7–23
γ-glutamyl transpeptidase (U/L)	12	9–32
Carcinoembryonic antigen (ng/mL)	<0.5	0–5
Carbohydrate antigen 19-9 (U/mL)	7.3	0–37

**Figure 3:**
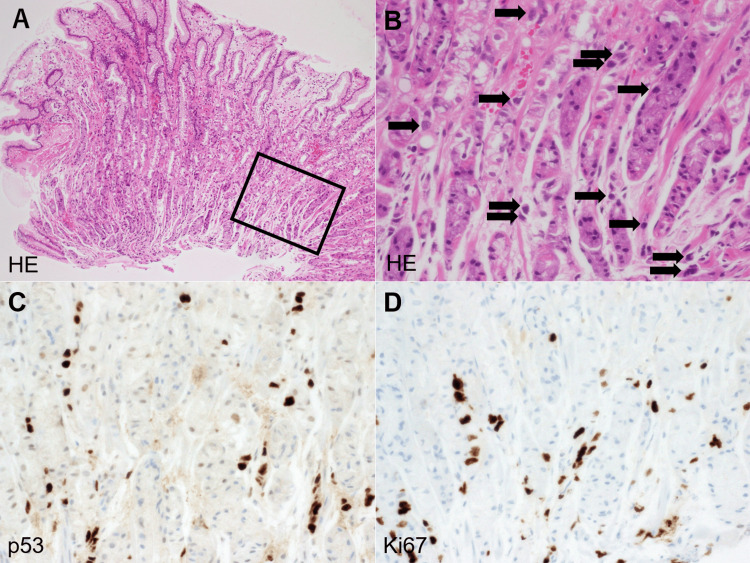
Histopathological and immunohistochemical findings of gastric biopsy specimens. Hematoxylin and eosin staining shows preserved fundic glands with edema and vascular congestion, but no apparent epithelial atypia (A). Immunohistochemical staining for p53 highlights scattered positive nuclei among the epithelial cells (B). Panel B is a higher magnification view of the boxed area in Panel A. p53 staining shows positive nuclear staining in tumor cells (C). Ki67 immunostaining also shows positive staining in scattered neoplastic cells, indicating a high proliferative index (D).

Initial histopathological evaluation showed preserved fundic gland mucosa with vascular congestion and edema but no evidence of carcinoma or epithelial atypia. Due to the strong clinical suspicion of malignancy, additional deep tissue sections and immunohistochemical staining were performed. On reevaluation, a small number of biopsy specimens revealed poorly differentiated adenocarcinoma and signet-ring cell carcinoma arranged in a sparse, single-cell pattern (Figure 3A, 3B). Immunostaining demonstrated positivity for p53 (Figure 3C) and Ki67 (Figure 3D), confirming the diagnosis of neoplastic cells.

The patient subsequently underwent total gastrectomy with Roux-en-Y reconstruction and cholecystectomy. Examination of the resected specimen revealed diffuse tumor infiltration throughout the stomach, consistent with scirrhous gastric cancer. Intraoperative peritoneal cytology was negative. Postoperatively, the patient began adjuvant chemotherapy with S-1 and docetaxel. After completing six courses of the adjuvant regimen, she was scheduled to continue oral S-1 monotherapy for six months. She has remained recurrence-free during the first postoperative year.

## Discussion

Scirrhous gastric cancer, also referred to as linitis plastica, is characterized by its diffuse infiltration into the submucosal and muscular layers of the stomach wall, often sparing the superficial mucosa. This histological growth pattern presents a significant diagnostic challenge, as tumor cells may be absent from the surface epithelium sampled during routine endoscopic biopsies. Previous studies have reported that conventional mucosal biopsies fail to detect malignancy in approximately 30-50% of cases, leading to delays in diagnosis and treatment initiation [3-5].

In the present case, the patient presented with non-specific symptoms such as epigastric pain and occasional postprandial vomiting, which prompted an esophagogastroduodenoscopy. The endoscopic findings were suggestive of an infiltrative process, including poor distensibility of the antrum and diffuse mucosal thickening features that had not been observed on the endoscopy performed seven years earlier. Despite these concerning macroscopic findings, the initial biopsy revealed no neoplastic cells. However, given the endoscopist’s high index of clinical suspicion, multiple biopsy specimens were obtained, and the pathologist was informed of the suspicion for scirrhous gastric cancer. Notably, the malignant cells were extremely sparse and subtle, even in deeper sections, and lacked overt cytological atypia. According to the pathologist, this was an exceptionally challenging case to diagnose based solely on hematoxylin and eosin staining, and the diagnosis might have been missed without immunohistochemical analysis and relevant clinical information. This underscores the crucial diagnostic role of p53 and Ki67 immunostaining in such elusive lesions, as well as the importance of close collaboration between endoscopists and pathologists, who must share critical clinical and histological information. Such interdisciplinary communication is essential in cases where typical histopathological features are not readily apparent. Previous reports have emphasized that repeat biopsy and supplementary immunostaining can improve the diagnostic yield, particularly when guided by strong clinical and endoscopic suspicion [6,7].

Regarding the diagnosis of gastric cancer, no clear recommendations on the exact number of biopsies are stated in guidelines or previous reports. However, in clinical practice, obtaining six to eight biopsies from different regions of the stomach is often recommended, especially when scirrhous gastric cancer is suspected. Repeat biopsies or the bite-on-bite technique (multiple biopsies from the same site) may improve the diagnostic yield. Although endoscopic ultrasound-guided fine-needle aspiration and submucosal tunneling biopsy have shown promise in enhancing diagnostic accuracy in scirrhous gastric cancer, with reported diagnostic yields exceeding 80% in some studies [5,8-11], they were not employed in this case. These techniques can be considered in scenarios where conventional biopsies are inconclusive, offering access to deeper layers of the gastric wall where tumor cells are more likely to reside [5,8].

## Conclusions

This case underscores the inherent diagnostic difficulty of scirrhous gastric cancer due to its subepithelial spread and the often normal appearance of the mucosa. More importantly, it highlights the pivotal role of meticulous endoscopic observation and proactive communication with the pathology team. A high index of suspicion, coupled with collaborative diagnostic efforts and the use of adjunctive tools when necessary, remains the cornerstone of accurately diagnosing this aggressive form of gastric cancer.
